# Vehicular Mini-LED backlight display inspection based on residual global context mechanism

**DOI:** 10.1007/s12200-024-00140-4

**Published:** 2024-10-29

**Authors:** Guobao Zhao, Xi Zheng, Xiao Huang, Yijun Lu, Zhong Chen, Weijie Guo

**Affiliations:** Department of Electronic Science, Xiamen University, Xiamen, 361000 China

**Keywords:** Mini-LED, Automated optical inspection, Deep learning, Display

## Abstract

Mini-LED backlight has emerged as a promising technology for high performance LCDs, yet the massive detection of dead pixels and precise LEDs placement are constrained by the miniature scale of the Mini-LEDs. The high-resolution network (Hrnet) with mixed dilated convolution and dense upsampling convolution (MDC-DUC) module and a residual global context attention (RGCA) module has been proposed to detect the quality of vehicular Mini-LED backlights. The proposed model outperforms the baseline networks of Unet, Pspnet, Deeplabv3+, and Hrnet, with a mean intersection over union (Miou) of 86.91%. Furthermore, compared to the four baseline detection networks, our proposed model has a lower root-mean-square error (RMSE) when analyzing the position and defective count of Mini-LEDs in the prediction map by canny algorithm. This work incorporates deep learning to support production lines improve quality of Mini-LED backlights.

**Graphical abstract:**

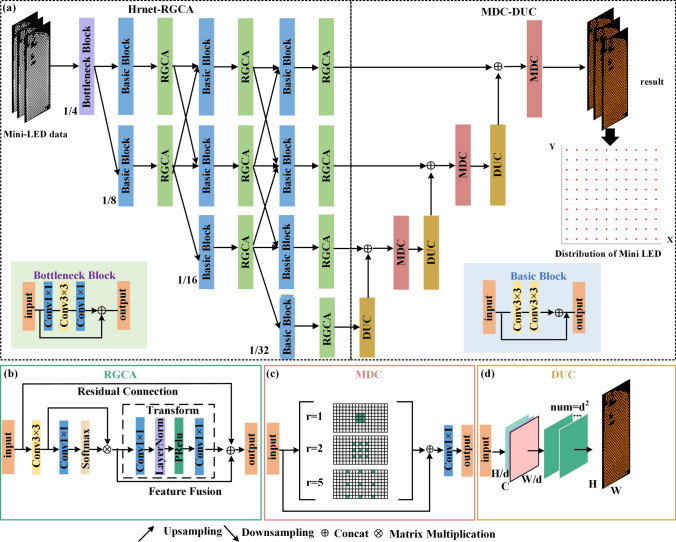

## Introduction

Recent advancements in LED technology have spurred the development of innovative lighting solutions, with Mini-LEDs gaining substantial attention [1–4]. Mini-LED is now a leading technology for automotive flat-panel displays [5], offering locally dimmable backlighting for high dynamic range (HDR) liquid crystal displays (LCDs) due to their superior brightness, enhanced contrast, and long operational lifespan [6–8]. For example, Akimoto et al. reported that a Mini-LED backlight can achieve over 5000 nits with excellent uniformity [9]. Yang et al. researched that Mini-LED backlit LCDs outperform OLED displays in certain applications [10]. Tan et al. analyzed the first Mini-LED backlit HDR LCD, showcasing significant improvements in image quality and contrast [11]. Particularly, in the manufacturing and integration of Mini-LED backlight panels, the inspection algorithms for individual Mini-LED chips are increasingly demanded. Nevertheless, the conventional inspection systems and manual inspection methods cannot meet the requirement of high-precision inspection for the compact Mini-LED layout.

With the development of optical measurement and image processing technologies, many endeavors have focused on the robust and accurate detecting for automated optical inspection (AOI) [12, 13], which has become an effective technique for detecting the chips in the Mini-LED backlight panel [14, 15]. The implementation of AOI in automated and smart manufacturing is more attractive than conventional inspection methods.

Deep learning acts as an effective tool for AOI applications and it can improve the efficiency of display quality inspection. For classification tasks, Nam et al*.* presented a framework for measuring and quantifying color defects by using HDR imaging and an image appearance model, aiming to improve the accuracy of automated inspection [16]. Chen et al*.* developed an automatic microscopic vision-based detection system and proposed the LBG-YOLO deep learning algorithm for Micro-LED defect detection [17]. Yang et al*.* proposed an online sequential classifier and transfer learning method for online training and classification of Mura defects. This method enables quick online defect classification of LED displays while costing fewer computational resources [18]. Park et al*.* proposed an efficient filtering method for classifying ambiguous surface defects on mobile display panels, using a random forest feature selection and real data from a smartphone display inspection line [19]. In addition, some segmentation methods have potential for applications in the display domain, such as Unet, Pspnet, DeeplabV3+, and high-resolution network (Hrnet) [20–24]. These segmentation models are effective at detecting and characterizing key features of displays, which could make them invaluable for improving display quality inspection. Hrnet has the advantage of maintaining high-resolution feature maps and integrating information at different scales at the same time, making it suitable for display performance inspection.

Building on these segmentation networks, attention mechanisms like squeeze-and-excitation (SE) block, convolutional block attention module (CBAM), and global context network (GCNet) could enhance control and precision in processing input data for display detection [25–27]. The core principle of the attention mechanism in the network is to prioritize the detection of specific regions of the input data. These mechanisms evaluate the significance of various areas or features, enabling the network to focus more on regions that are crucial for the current task, such as dead pixels within a display. This targeted method complements the application of advanced convolutional techniques, which increase the accuracy of display detection. For instance, depth-wise convolutions, grouped convolutions, and dilated convolutions all play important roles in enhancing detection capabilities [28, 29]. The incorporation of several convolutional approaches improves the capacity of networks to recognize or segment relevant patterns and regions in input data, increasing the precision of complicated detection and segmentation tasks.

When evaluating display quality, the distribution of the LED chip is a critical component that determines display performance [30, 31]. The distribution of Mini-LED chips is of paramount importance in ensuring the overall visual effect of the screen. Inaccuracy in the positioning of the chips may result in some adverse effects, including uneven brightness, color distortion, or even localized shadowing. Therefore, employing AOI technology to accurately measure and analyze the positions of LED chips is vital for enhancing display quality and ensuring consumers enjoy a high-quality visual experience. The applications of display detection primarily focus on detecting dead pixels and LED positioning through deep learning techniques. Mini-LED chips are densely distributed, and even a small deviation in LED position or brightness can seriously affect display quality. The lack of Mini-LED backlight sample data also restricts the development of detection algorithms using deep learning and pattern recognition, as these technologies require extensive data on various defect types to enhance detection accuracy and reliability.

To meet these challenges, various dead pixel scenarios for Mini-LED backlights have been simulated with baffle plate in this work. For detection model, mixed dilation convolution dense upsampling convolution (MDC-DUC), and the residual global contextual attention (RGCA) module have been introduced to Hrnet. The MDC-DUC enhance the receptive field of the network, enabling the effective featuring of Mini-LED locations at different scales. Moreover, RGCA allows the network to focus on the luminous region of each Mini-LED, improving the detection efficiency of small differences. Furthermore, the model enables the AOI device to detect individual Mini-LED regions with precision, thereby facilitating the efficient acquisition of the position and defective counts of Mini-LEDs on the backlight panel. This work can provide guidelines and effective solutions for the quality control of the manufacturing of mini-LED backlight.

## Method

**Fig. 1:**
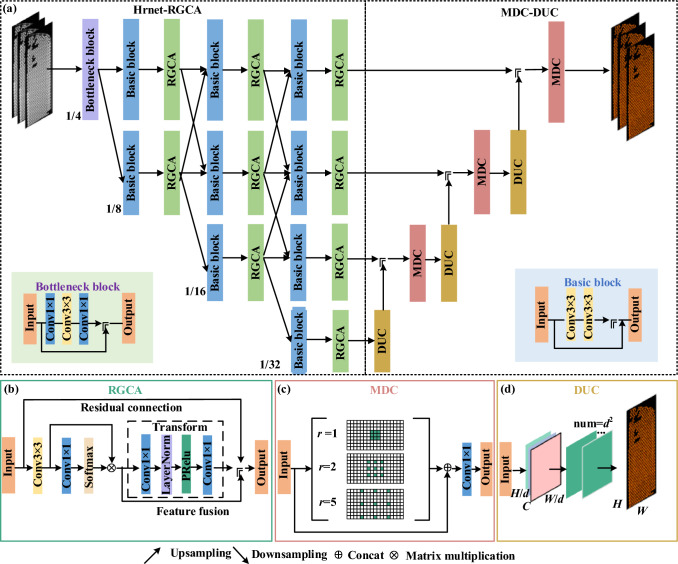
Schematic of proposed model structure: **a** the entire structure, **b** RGCA, **c** MDC, and **d** DUC

As illustrated in Fig. 1, our proposed network architecture comprises RGCA and MDC modules, which are incorporated into Hrnet to detect Mini-LEDs backlights. The global context-aware capability of RGCA is compatible with handling densely distributed small targets, thus improving the ability to detect these small targets that can be easily misclassified as background. The parallel feature maps of the Hrnet preserve high-resolution features and enhance accurate prediction of blurry boundaries, while the MDC module broaden the receptive field and improves the detection of Mini-LED with different scales. Moreover, particularly for small Mini-LEDs with boundaries, the DUC improves the capture of individual details. The proposed approach allows for accurate semantic segmentation of Mini-LEDs by smoothly fusing multi-target feature maps.

### High-resolution network backbone

As shown in Fig. 1a, the primary framework used in this research is the Hrnet-RGCA backbone network, consisting of two key components: the basic block and the bottleneck block. The basic block is composed of two 3 × 3 convolution layers, where each layer applies a 3 × 3 kernel to the input feature map. This allows the model to capture localized spatial information efficiently. Each 3 × 3 convolution is followed by a batch normalization layer, which stabilizes the learning process, and a ReLU activation function, which introduces non-linearity, enabling the model to learn more complex patterns effectively. This structure ensures efficient feature extraction while maintaining the spatial resolution of the input.

The bottleneck block is designed to balance computational efficiency and depth. It begins with a 1 × 1 convolution layer, which reduces the number of feature channels (i.e., dimensionality reduction) without altering the spatial structure of the feature map. This step is crucial in lowering computational costs while preserving important information. After that, a 3 × 3 convolution layer is used to capture spatial features, and finally, another 1 × 1 convolution layer is applied to restore the dimensionality of the feature map. Similar to the basic block, each convolution in the bottleneck block is followed by batch normalization and a ReLU activation function. The 1 × 1 convolutions are particularly important for managing the dimensionality, ensuring the network remains efficient while maintaining strong feature representation capabilities. Hrnet adopts a parallel structure that processes feature maps at different resolutions simultaneously, ensuring that high-resolution details are maintained throughout the network. This parallelism is essential for tasks involving objects with unclear boundaries or fine details. Finally, the RGCA module enhances the capability of network by capturing both global context and fine-grained details, further improving segmentation accuracy.

### Residual global context attention module

As shown in Fig. 1b, RGCA contributes to the efficient detection and segmentation of densely distributed chips on a mini-LED backlight panel. In the *n*th residual group, the formula of the RGCA is described as:

1 $$ F_{n,m} = F_{n,m - 1} + H_{n,m} (X_{n,m} ), $$

where *F*_*n*,*m*−1_ and *F*_*n*,*m*_ are the input and output of RGCA, respectively. *H*_*n*,*m*_ represents the global-context attention function applied to *X*_*n*,*m*_, which is the result of applying a convolution to *F*_*n*,*m*−1_. The global-context attention encompasses context modeling, feature transformation, and fusion, expressed as:

2 $$ H_{n,m} (X_{n,m} ) = H_{F} \left( {X_{n,m} ,\phi \left( {\sum\limits_{j}^{{N_{{\text{p}}} }} {\psi^{j} X_{n,m}^{j} } } \right)} \right), $$

the context modeling process in Eq. (2) is crucial for capturing global spatial relationships within the feature map. Specifically, the term $\sum\nolimits_{j}^{{N_{{\text{p}}} }} {\psi^{j} X_{n,m}^{j} }$ represents the aggregation of information from multiple spatial positions *j* across the feature map *X*_*n*,*m*_, where *N*_p_ denotes the total number of positions, and *ψ*^*j*^ acts as the weight for each position. This weighted summation enables the network to capture long-range dependencies by assigning different levels of importance to various positions in the feature map. The ability to model context beyond local pixel neighborhoods is particularly beneficial for tasks involving densely distributed targets, such as detecting mini-LED chips. By incorporating global contextual information, the model can better understand how different regions relate to one another, leading to more accurate detection. After the context is aggregated, the function *ϕ*[…] performs feature transformation, which refines the gathered information through operations like convolution and activation. This transformed context is then integrated into the original feature map via a residual connection, ensuring that both local details and broader spatial relationships are retained. The RGCA was integrated into Hrnet pathways to augment features with global context information, enhancing the accuracy of Mini-LED chip localization and segmentation.

### MDC-DUC module

To enhance optimization in the perceptual domain, the MDC-DUC module has been integrated into the feature fusion stage. Figure 1c illustrates a schematic representation of dilated convolutions with varying dilation rates (e.g., *r* = 1, 2, 5) from the MDC block, resulting in typical convolutional receptive fields of 3, 5, or 11. The dilated convolution operation is defined by the following formula:

3 $$ r^{\prime} = d \times (r - 1) + 1, $$

where *r* denotes the size of the input convolutional kernel, *d* represents the dilation factor, and *r′* signifies the size of the convolutional kernel following dilation. Calculating the output dimension of the convolutional layer is presented as follows:

4 $$ o = \left\lfloor {\frac{i + 2p - r^\prime }{t}} \right\rfloor + 1, $$

where *i* stands for the dimensions of the input feature map, o for the dimensions of the output feature map, *p* for the size of pooling, *t* for the stride, and ⌊…⌋ indicates rounding-down operation. Gridding effects of mixed dilated convolutions are minimized while this design maximizes information collection across sizes. A ResNet-inspired skip connection strategy, incorporating concatenation operations between mixed convolution outputs and input feature maps, and 1 × 1 convolutions, is utilized for efficient feature fusion. In this way, comprehensive processing of high-resolution characteristics is provided, which is crucial for Mini-LED backlight detection, and the collection of semantic information is expanded.

According to Fig. 1d, the DUC module replaces bilinear interpolation in recovering image dimensionality. The DUC processes images *X* of dimensions* R*^*C*×*H*×*W*^, where *C, H,* and *W* are channel count, height, and width, respectively. This module outputs an *H* × *W* label map, assigning each pixel a category label. The feature map from the MDC block, *x* with dimensions *R*^*c*×*h*×*w*^ (*h* = *H*/*d, w* = *W*/*d, d* being the sampling factor), is reshaped by DUC. First, it transforms *x* into *x′* of dimensions *R*^(*d*×*c*×*d*)×*h*×*w*^ through a 3 × 3 convolution. Then, *x′* is reshaped to *x′′* of dimensions *R*^*c*×(*h*×*d*)×(*w*×*d*)^, maintaining the height *H*, width *W*, and channel *C* of the images. A direct convolution operation on the hybrid dilation convolution of the DUC output yields *H* × *W* data. By upsampling and fusing three smaller feature maps and their DUCs on a layer-by-layer basis, the method improves detail and output quality.

## Experiment

**Fig. 2:**
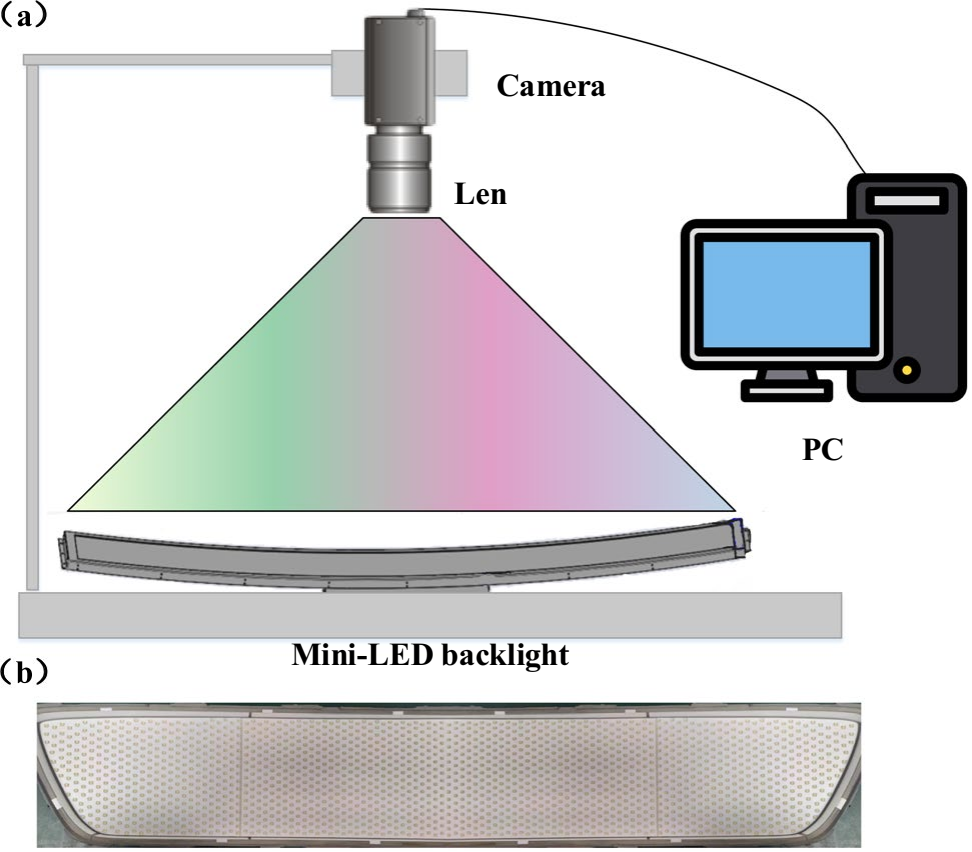
System and sample: **a** schematic diagram of the experimental setup for data acquisition using a CCD camera, with data transmission to a PC for analysis; **b** physical photograph of the Mini-LED backlight panel sample

As depicted in Fig. 2a, data acquisition is conducted using a CCD camera model (MV-CS050-10GM) paired with a 12 mm industrial lens (MVL-M), which achieves a resolution of 2448 × 2048 pixels. The pixel size is 3.45 µm × 3.45 µm, and the sensor format measures 2/3 inches. The CCD camera captures high-resolution images of the Mini-LED backlight panel, which are then transmitted directly to a PC for further analysis and processing. This setup ensures precise data acquisition, which is crucial for evaluating the performance of the Mini-LEDs. According to Fig. 2b, The Mini-LED backlight panel used in this study has individual Mini-LED chips measuring about 100 μm × 200 μm, with a spacing of 850 μm. This backlight panel features a curvature from 4 to 5 degrees in a non-standard configuration. The experimental sample in this study is a Mini-LED backlight panel approximately 76 cm in length and 11.8 cm in width. The backlight panel consists of 1733 Mini-LEDs, divided into 869 chips on the left panel and 864 chips on the right panel.

**Fig. 3:**
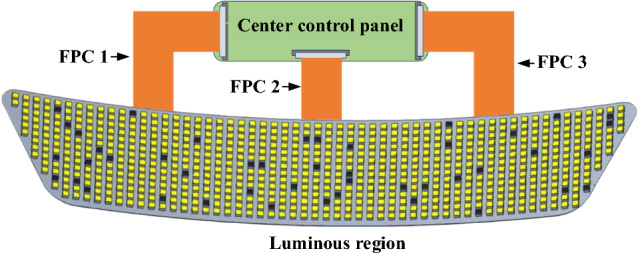
Schematic diagram illustrates the Mini-LED display

Figure 3 shows the Mini-LED example circuit control structure design, the main control board contains the timing controller (TCON) specialist IC, flash storage integrated circuit (IC), power IC, and others. TCON processes embedded display port (EDP) video signals from an external host computer. It deciphers, compensates, and merges the backlight serial peripheral interface (SPI) signal into the EDP signal internally. Flexible printed circuit (FPC) 1 controls power on the left and FPC3 controls EDP output on the right. The main control board sends each picture frame to the Mini-LED via the FPC2 EDP signal and the SPI signal to the backlight board, performing central control and processing. In Fig. 3, the yellow squares indicate that the Mini-LEDs are illuminated, and the black squares show the defective Mini-LEDs that we simulated by randomly covering them with customized light baffles.

**Fig. 4:**
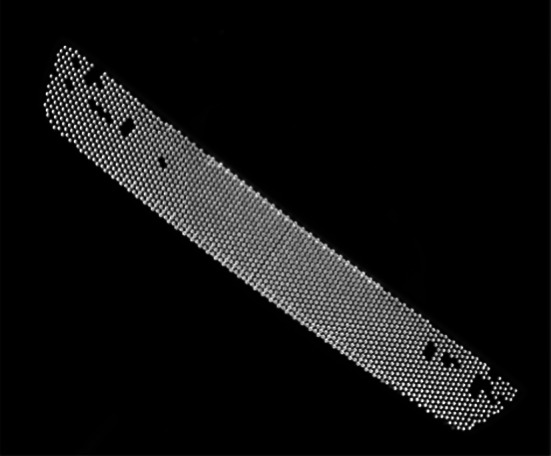
Image of randomly obstructed lighting Mini-LED backlit panel

Simulations were conducted under different luminous conditions of multiple Mini-LED backlight panels, and a customized backlight panel was used to randomly obstruct the Mini-LEDs on the backlight panels in this study. One of the simulation results is shown in Fig. 4. This approach enables the collection of 2118 distinct images, each showcasing different illuminated areas of the LED panel. For improving the objectivity and accuracy of deep learning labeling, the Segment Anything model combined with Labelimg is employed. This methodology involved the creation of masks for each Mini-LED light-emitting area on the Mini-LED backlight panel, ensuring objective and precise labeling for subsequent analysis.

To validate the effectiveness of the proposed improvements to Hrnet, an ablation study was conducted on the Mini-LED vehicular backlight panel dataset. In our experiments, the dataset was divided into 90% for training and 10% for validation. Our model training employed the original Hrnet as the baseline model, which was trained from scratch. The model was compared with several popular semantic segmentation networks, including Pspnet, Unet, Deeplabv3+, and the original Hrnet, with each model trained for over 1000 epochs. The experiments were conducted using a training environment equipped with 3* NVIDIA GTX 1080 Ti GPUs.

**Fig. 5:**
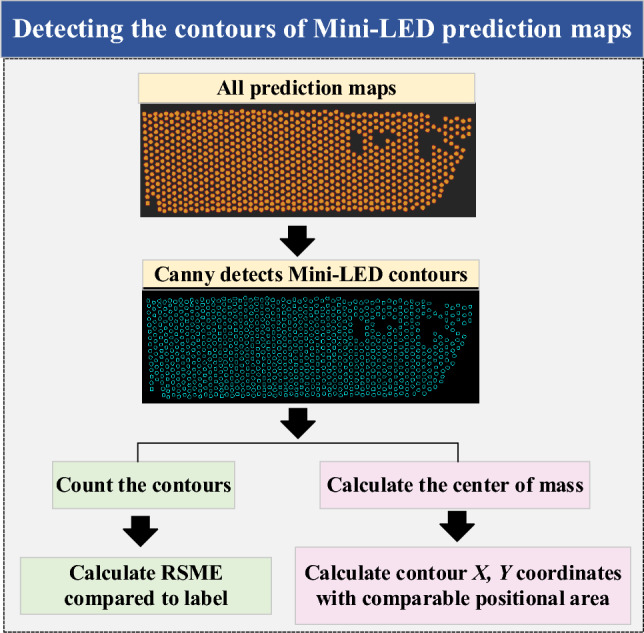
Flowchart of the detection model to predict Mini-LED contours

This work employs the binary segmentation outputs from these models, thereby eliminating the need for grayscale conversion and noise reduction. Edge detection was performed using the canny algorithm for the Mini-LEDs of the prediction maps of each model, according to the pairs shown in Fig. 5. Contours of the illuminated Mini-LED areas can be directly extracted from these binary outputs of the networks. In the extraction process, an empirical threshold is utilized to identify luminous Mini-LEDs, evaluating potential discrepancies in LED counts across various models and backlight panels.

5 $$ D = T - N, $$

where *D* represents the number of defective Mini-LEDs, *T* is the total number of Mini-LEDs on the panel, and *N* is the number of non-defective Mini-LEDs identified.

The root mean square error (RMSE) was calculated to assess the count of defective Mini-LEDs and the position of functional ones across backlight boards in 2118 different masking scenarios:

6 $$ {\text{RMSE}}_{{{\text{count}}}} = \sqrt {\frac{1}{n}\sum\limits_{i = 1}^{n} {(D_{i} - L_{i} )^{2} } } , $$

7 $$ {\text{RMSE}}_{{{\text{postion}}}} = \sqrt {\frac{1}{n}\sum\limits_{i = 1}^{n} {(P_{i} - L_{i} )^{2} } } , $$

where *n* is the number of Mini-LEDs, *D*_*i*_ represents the number of defective Mini-LEDs detected. *P*_*i*_ represents the predicted centroid position for the *i*th Mini-LED, and *L*_*i*_ is the label. RMSE_count_ calculates the error of defective LEDs and labels on the backlight board. The RMSE_position_ calculation excludes contours without a ground truth label, ensuring that only relevant deviations affect the accuracy metric.

## Results and discussion

In evaluating the performance of the semantic segmentation models, three primary metrics are utilized: mean intersection over union (Miou), mean pixel accuracy (mPA), and Accuracy. These metrics are calculated as follows:

8 $$ {\text{Miou}} = \frac{1}{n}\sum\limits_{i = 1}^{n} {\frac{{{\text{TP}}_{i} }}{{{\text{TP}}_{i} + {\text{FP}}_{i} + {\text{FN}}_{i} }}} , $$

9 $$ {\text{mPA}} = \frac{1}{n}\sum\limits_{i = 1}^{n} {\frac{{{\text{TP}}_{i} }}{{{\text{TP}}_{i} + {\text{FN}}_{i} }}} , $$

10 $$ {\text{Accuracy}} = \frac{{\sum\nolimits_{i = 1}^{n} {{\text{TP}}_{i} } }}{{{\text{TNP}}}}, $$

where *n* is the number of segmentation classes in the dataset, TNP is total number pixels of input map, TP_*i*_ represents the number of pixels correctly identified as belonging to class *i*; FP_*i*_ refers to the number of pixels incorrectly labeled as class *i* but actually belonging to a different class, and FN_*i*_ denotes the number of pixels that belong to class *i* but are not detected by the model. These parameters are foundational for the metrics used to assess the accuracy of the model in segmenting different classes and minimizing classification errors.

According to the data in Table 1, our proposed model has shown improvements in key evaluation metrics. Compared with the original Hrnet and other baseline models, our model performed better in Miou, mPA, and Accuracy, reaching 86.91%, 92.45%, and 98.04% respectively. These results demonstrate that adjusting multiscale processing and edge refinement produced some outcomes. When compared with other models such as Unet and Pspnet, the proposed model has demonstrated a moderate performance enhancement. Although Deeplabv3+ was initially better in the mPA metric than original Hrnet, the proposed model improved slightly following improvements. In this model comparison, we emphasize the performance of our proposed network relative to the original Hrnet. One important factor we considered is latency, which measures the forward propagation time per image and serves as a key metric for evaluating operational efficiency. While Hrnet achieves a latency of 1.43 s per image, our model closely matches this with a latency of 1.47 s per image. Although we increased the complexity of the network, the prediction time on the GPU remains nearly comparable to that of the original network, while achieving higher accuracy. This demonstrates that our modifications not only maintain a competitive Latency but also lead to enhanced precision. The ability to achieve better accuracy with a Latency similar to that of the original Hrnet highlights the effectiveness of our improvements, making our model an optimal choice for applications requiring both efficiency and high performance.

**Table 1:** Comparison of model test results

Model	Miou	mPA	Accuracy	Latency
Unet	80.87%	88.26%	95.25%	1.83 s
Pspnet	65.64%	84.99%	89.22%	1.66 s
Deeplabv3+	84.89%	91.76%	96.86%	1.54 s
Hrnet	85.86%	91.19%	97.13%	1.43 s
Ours	86.91%	92.45%	98.04%	1.47 s

**Fig. 6:**
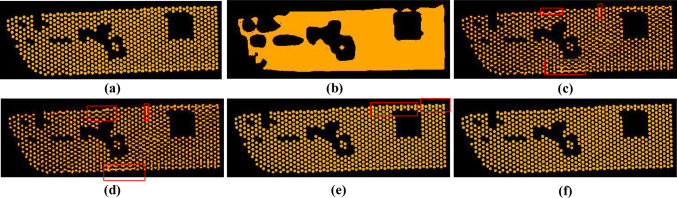
Visual comparisons for our proposed model and baseline models: **a** label, **b** Pspnet, **c** Unet, **d** Deeplabv3+, **e** Hrnet, and **f** our proposed model

As shown in Fig. 6, a visual evaluation has been performed among the baseline model and the proposed model. The regions marked by red rectangles indicate the inaccurately predicted Mini-LED regions in which the features are recognized as connected units. As illustrated in Fig. 6b, the Pspnet cannot segment each Mini-LED in some situations. In Fig. 6c–e, the predictive maps generated by Unet, Deeplabv3+, and the original Hrnet exhibit varying degrees of contour inaccuracy, with multiple Mini-LED illumination contours that could not be individually distinguished, and contour concatenation, suggesting that these models have limitations in accurately segmenting individual Mini-LEDs. Our proposed model has better detection of Mini-LED light emission contours, which are very similar to labels.

**Fig. 7:**
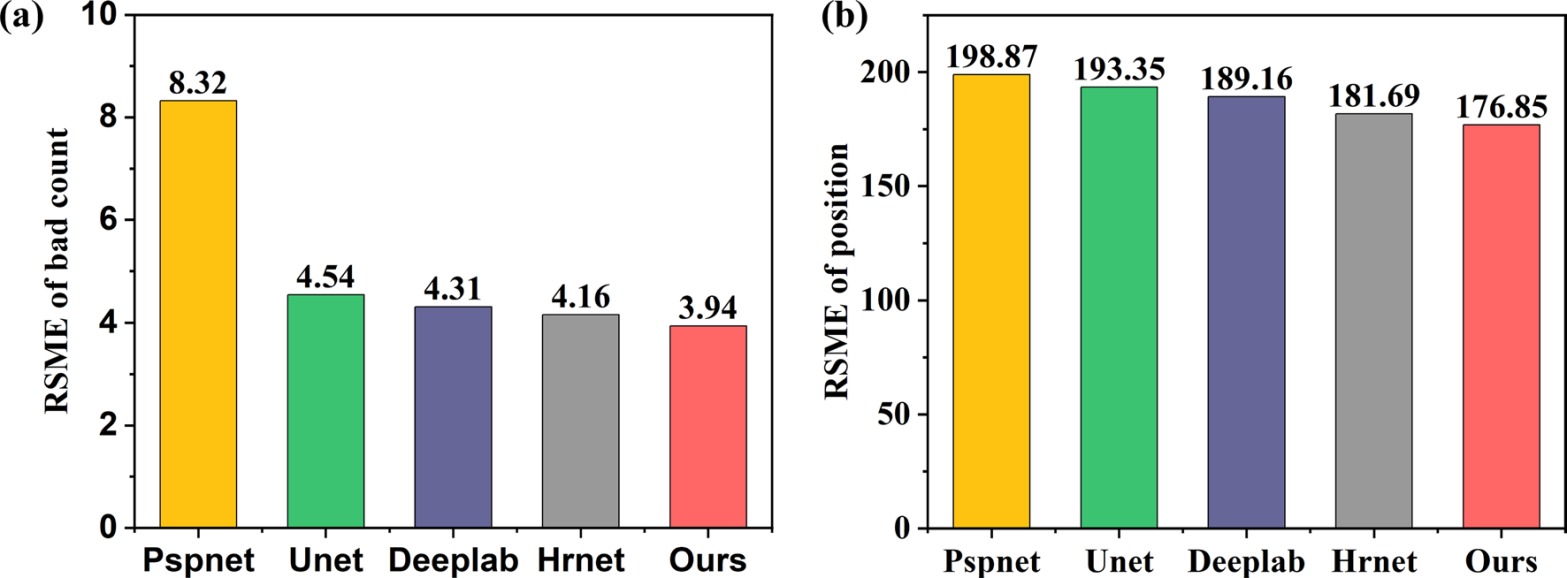
Histogram of RMSE: **a** RMSE for defective count of Mini-LED contours predicted, **b** RMSE for the position of Mini-LED contours predicted

Subsequently, the technique of canny calculus was employed to identify the boundaries of the contours of Mini-LEDs as predicted by each model, with the objective of calculating RMSE_count_ and RMSE_position_*.* Figure 7a depicts the RMSE_count_ of the Mini-LED contour for different model prediction maps. Compared to the limiting model, our proposed model obtains the lowest root mean square error of 3.94, which reflects our higher accuracy in calculating the actual number of Mini-LEDs per panel. Accurate count assessment is pivotal for ensuring the luminance consistency across the display, directly impacting the visual performance of the final device. Figure 7b shows the RMSE_position_ of the Mini-LED contour for different model prediction maps. Even after using Canny to detect the center-of-mass position of the predicted map, our model still exhibits the lowest positional error with an RMSE value of 176.85 among all models in our experiments. This result shows that our model exhibits higher accuracy in reproducing the true distribution of Mini-LEDs on the backlight panel.

## Conclusions

Based on our research and experimental results, we have studied the effectiveness of integrating MDC-DUC and RGCA modules into Hrnet for detecting Mini-LED chips. We evaluated the model’s performance in terms of accuracy, precision, and robustness in identifying dead pixels and chip placement. We also addressed data augmentation by simulating various dead pixel scenarios and provided a quantitative analysis using Canny edge detection. The following specific conclusions can be drawn:

1. The integration of MDC-DUC and RGCA modules into Hrnet significantly improves the ability to accurately detect Mini-LED chips. This enhanced model demonstrates superior precision in identifying dead pixels and chip placement. Our model outperforms baseline models in Miou, mPA, and Accuracy, achieving 86.91%, 92.45%, and 98.04%, respectively.
2. By simulating various dead pixel scenarios using a baffle plate, we effectively augmented our dataset. This approach addresses the critical issue of limited data availability, which is essential for training robust detection models.
3. Utilizing the Canny edge detection algorithm, we analyzed and evaluated the position and defect count of Mini-LEDs. This method provides quantitative measurements, achieving a RMSE of 3.94 for defect count and an RMSE of 176.85 for position, which further optimizes the evaluation of dead pixels and chip placement on Mini-LED backlight boards.

## Data Availability

The raw or processed data required to reproduce these findings cannot be shared, as the data also forms part of an ongoing study.
